# Supplementary material for: Autumn shifts in cold tolerance metabolites in overwintering adult mountain pine beetles
Source: PLoS One. 2020 Jan 8;15(1):e0227203. doi: 10.1371/journal.pone.0227203 (PMC6948739; doi:10.1371/journal.pone.0227203)
Supplement: Attachment — Submitted filename: Response to Reviewers.docx [file pone.0227203.s003.docx]

Response to Reviewers

Dear Dr. Bone,

Please find below our response to the reviewer’s comments on our original manuscript. We have addressed their reviews comment by comment where possible, in order to more clearly indicate our response to each point.

Reviewer 1:

1) Ln. 24, 112: Revise to “…nuclear magnetic resonance (NMR).”

Revised as suggested.

2) Ln. 26: Revise to “…beetles appear to prepare…”

Revised as suggested.

3) Ln. 27-28: This statement is not substantiated by the data as concentrations of metabolites were not analyzed for larvae. Review and revise as appropriate throughout the ms.

Revised to: “…the same cold-tolerance compounds found in other insect larvae including mountain pine beetle…” Other studies referenced within the paper have found cryoprotectants in conspecifics and other species of overwintering beetles.

4) Ln. 73: Replace “campground” with “Campground”

Revised as suggested.

5) Ln. 75: Delete “is located very near to the Great Divide”; redundant w/ ln. 93.

Revised as suggested.

6) Ln. 81: Define how it was determined these were “new adult beetles” vs. re-emergent adults.

Revised to add: “New adults were identified based on their proximity to larval galleries and their relative size.”

7) Ln. 95: Revise to “Mountain pine beetle larvae…”

Revised as suggested.

8) Ln. 144: Was this the “coldest temperature of the winter” (12/21–3/20) or during the collection period? Review and revise as appropriate.

Revised to “the coldest on-site temperature of the winter.” By the data on our climate loggers, it was the coldest day in between Sept 2016-May 2017, therefore the coldest day that the studied beetles experienced.

9) Ln. 179-180: Don’t these statements largely reflect the same condition (i.e., snow insulates within bark temperatures)? Review and revise as appropriate.

Revised to: “Mountain pine beetle larvae typically survive winters if ambient temperatures do not fall below approximately –40℃, though larvae insulated below the snow line may survive these cooler conditions.”

10) Ln. 196: Provide complete contact information for Batista.

Revised as suggested.

11) Ln. 231: Revise to “…they do not…”

Revised as suggested.

12) Ln. 234: Revise to “As ice crystals were observed within galleries on the coldest sampling day, this may have been…”

Revised as suggested.

13) Ln. 243: Revise to “…likely making…”

Revised as suggested.

14) Ln. 245-246: Revise to “…mountain pine beetle adults produce three…”

Revised as suggested.

15) Ln. 250: Replace “park” with “Park”

Revised as suggested.

16) Ln. 251 (and throughout): Delete “MPB” or introduce at first mention of mountain pine beetle and carry throughout the ms.

Revised as suggested.

17) Ln. 253-255: Delete sentence.

Revised as suggested.

18) Ln. 255: Does the length of the winter matter? Revise.

Revised to: “In areas where winters begin earlier in the year, thus reducing the cold acclimation period,”. We revised this to speak to how soon the winter starts. Rapid onset of winter after warmer summer temperatures reduces the period of time available for beetles to produce necessary cryoprotectants.

19) Ln. 256: Revise to “…-30 ℃ for several days…”

Revised as suggested.

20) Ln. 260: Do you expect similar results in jack pine? Elaborate.

Revised to: “This work provides new parameters for modeling the spread of mountain pine beetles in their expanding geographic range and is evidence of physiological plasticity in this insect. In both novel, colder regions like the Boreal Forest and warmer ecosystems which experience more developmental degree days, we may see the effect of natural selection amplifying varied life cycle lengths (longer or shorter) in mountain pine beetle.”

This expands our original point to include possible outcomes in the Jack pine forests of the Boreal forest.

21) Figure 2: Add dates to X-axis.

Revised as suggested.

Reviewer 2:

1)This is a well-written paper presenting important new information on the overwintering ecology of mountain pine beetle. The overwintering capability of adult beetles has been scantly examined.
The paper is worthy of publication because of the new findings of cryoprotectans in adult beetles.
Although I am recommending a minor revision there are two items that the authors should address.
First, I am always hesitant to accept findings from a field study of this type with only one year of data. A minimum of two years at least allows some level or repeatability. Environmental factors, specifically in this case temperature, can influence findings in unpredictable ways. The authors acknowledge the possible influence in lines 222-225 where they indicate “While field temperatures initially dropped at a steady rate from September to early October, they rewarmed between mid-October and November (Fig 3). The extended period of warmer weather may have confused the cues that normally trigger the beetles to produce cryoprotectants.” I would offer that the authors consider offering some justification and support as to why data from only one year can be considered reliable.

Our response: While we acknowledge that we have one year of data, the nature of new adult MPB makes them difficult to find and unlikely to recur in the same areas within a reasonable time frame for collection and study. We feel that this data is important because it demonstrates that these beetles retain the capacity to generate cryoprotectants even though they are no longer larvae, a finding that has never been documented before.

2) Second, throughout the discussion the authors refer to “increased mortality rates” (of beetles) or statements along these lines. Mortality was not quantified (as far as I can tell). The statement seems to refer to the observation of no live beetles in their last sampling date. To infer increased mortality rates by examining 5 trees in a single date does not seem appropriate. Within a small area, even in the same stand it is possible to find a few trees without surviving beetles and trees with surviving beetles. I would recommend that the authors remove this from the discussion.

Revised to: “A spring collection was attempted when the site was again accessible following snow melt on 26 May 2017, but no living new adults could be found in a search of the area, including thorough investigation of trees previously sampled (number of trees checked on site > 50).”

Also important – on Line 95 in the methods, the paper talks about larvae being processed. Is this correct? Or is this supposed to be adults?

Revised to “beetles” throughout methods section.

Line 35, suggest remove the comment of “unprecedented” as other studies are showing that this is unlikely the case.

Revised to “unusual.”

Line 39, although this is well established, think it is always good to have a link to a weather data website of a citation that provides data.

Revised to add citation.

Line 47, are the beetles really freeze intolerant? They have evolved a biological mechanism to withstand freezing temperatures.

Yes, they are intolerant to freezing, though capable of surviving freezing temperatures above the -40℃ threshold. Mountain pine beetles generate cryoprotectants to reduce their freezing point, but the formation of internal ice crystals causes too much cell damage for survival upon thawing. see Bentz BJ, Mullins DE. Ecology of mountain pine beetle (Coleoptera: Scolytidae) cold hardening in the Intermountain West. Physiol Chem Ecol. 1991;28: 577–587. doi:10.1093/ee/28.4.577

Line 37, are these air or subcortical temperatures? Likely do not differ too much but would be good to clarify.

Revised to specify air temperatures

Lines 90-91, this sentence is confusing, please clarify.

Revised to: “Climate loggers were moved from their original positions after the eighth sampling day and relocated to other trees near to their original positions within the sampling area due to sanitation logging removing the original trees used for placement.”

Line 112, please spell out NMR so the unfamiliar reader can understand or at least search what the technique is.

Revised, NMR is now spelled out earlier in the paper as per reviewer 1’s request.

Figure 2, again – air temperatures?

Revised to: “ambient air temperatures.”

Line 153, from Figure 3, seems like 3 compounds were elevated as much as Proline – are these not important? Should these be discussed? What are they?

The figure 3 graph shows log transformed p-values for the anova and not the actual level of metabolite found, hence a higher value does not mean metabolites are present at ‘elevated’ levels, but only show when the values differed significantly at any point within the study. Some compounds detected in our study were statistically significant but not biologically significant due to very, very minute changes in actual metabolite concentration. Due to the nature of cryoprotectants, values at the ends of the detection limit of our methods would most likely not have any noticeable effect on prevention of freezing.

Figure 4, may be better to have the actual sampling dates in the x-axis.

Revised as suggested.

Line 160 and Figure 5, not sure I follow this – negative correlations but the line is increasing. The x-axis goes from -10 to 20 C but the x-axis label indicates neg Celsius. Are the numbers on the x-axis incorrect?

Revised to add more information about the negative transformation of temperature in the methods section. This information is also referenced in the caption for Figure 5.
